# Identification of a biosynthetic gene cluster for a red pigment cristazarin produced by a lichen-forming fungus *Cladonia metacorallifera*

**DOI:** 10.1371/journal.pone.0287559

**Published:** 2023-06-23

**Authors:** Jaycee Augusto Gumiran Paguirigan, Jung A. Kim, Jae-Seoun Hur, Wonyong Kim

**Affiliations:** 1 Korean Lichen Research Institute, Sunchon National University, Suncheon, Korea; 2 Department of Biological Sciences, College of Science, University of Santo Tomas, Manila, Philippines; University of Nebraska-Lincoln, UNITED STATES

## Abstract

Lichens are known to produce many novel bioactive metabolites. To date, approximately 1,000 secondary metabolites have been discovered, which are predominantly produced by the lichen mycobionts. However, despite the extensive studies on production of lichen secondary metabolites, little is known about the responsible biosynthetic gene clusters (BGCs). Here, we identified a putative BGC that is implicated in production of a red pigment, cristazarin (a naphthazarin derivative), in *Cladonia metacorallifera*. Previously, cristazarin was shown to be specifically induced in growth media containing fructose as a sole carbon source. Thus, we performed transcriptome analysis of *C*. *metacorallifera* growing on different carbon sources including fructose to identify the BGC for cristazarin. Among 39 polyketide synthase (PKS) genes found in the genome of *C*. *metacorallifera*, a non-reducing PKS (coined *crz7*) was highly expressed in growth media containing either fructose or glucose. The borders of a cristazarin gene cluster were delimited by co-expression patterns of neighboring genes of the *crz7*. BGCs highly conserved to the cristazarin BGC were also found in *C*. *borealis* and *C*. *macilenta*, indicating that these related species also have metabolic potentials to produce cristazarin. Phylogenetic analysis revealed that the Crz7 is sister to fungal PKSs that biosynthesize an acetylated tetrahydoxynaphthalene as a precursor of melanin pigment. Based on the phylogenetic placement of the Crz7 and putative functions of its neighboring genes, we proposed a plausible biosynthetic route for cristazarin. In this study, we identified a lichen-specific BGC that is likely involved in the biosynthesis of a naphthazarin derivative, cristazarin, and confirmed that transcriptome profiling under inducing and non-inducing conditions is an effective strategy for linking metabolites of interest to biosynthetic genes.

## Introduction

Lichen-forming fungi, with about 20,000 known species, form symbiotic relationships with algae and/or cyanobacteria [1]. Lichen thalli contain many bioactive compounds with pharmacological properties including antimicrobial, anti-proliferative, cytotoxic, and antioxidant activities [2–6], which are mainly produced by lichen-forming fungi, called mycobiont [4]. Secondary metabolites in lichens include the structurally diverse aromatic polyketides, which are biosynthesized through the successive condensation of acetyl units derived from malonyl-CoA [7–9]. The biosynthetic enzymes involved in the synthesis of aromatic polyketides, such as depsides and depsidones, are non-reducing polyketide synthases [9–12].

Cultivated mycobionts isolated from *Cladonia cristatella* and *C*. *metacorallifera* biosynthesize aromatic polyketides, cristazarin and 6-methylcristazarin, which are naphthazarin derivatives [13, 14]. Naphthazarin (5,8-dihydroxy-1,4-naphthoquinone) is derived from naphthoquinone through replacement of two hydrogen atoms with hydroxyl groups. Naphthazarin is a naturally occurring 1,4-naphthoquinone derivative, and is biosynthesized by bacteria, fungi and many plants including several species of Bignoniaceae, Boraginaceae, Droseraceae, Juglandaceae, and Plumbaginaceae [15]. A naphthazarin derivative, cristazarin, exhibited significant biological properties, including antibacterial, antitumor, and anticancer activities [13, 14]. Based on the genome sequence of *C*. *metacorallifera*, a total of 30 iterative type I polyketide synthase (PKS) genes were predicted [16]. However, the required biosynthetic gene cluster (BGC) for the biosynthesis of cristazarin is uncharacterized and the precise biological pathway still needs to be elucidated.

Due to the increasing availability of genomic resources [17, 18], and natural product databases in fungi [19], *in silico* approaches for linking secondary metabolites with their respective BGCs are becoming more common [20–25]. BGCs are a locally clustered group of genes that together encode a biosynthetic pathway for a compound [26, 27]. A BGC may include genes encoding biosynthetic enzymes, regulatory factors, and transporters. The core biosynthetic enzyme, such as PKS, synthesizes the backbone of the molecule, which is subsequently modified by various tailoring enzymes to produce the final products [4, 26, 28]. Major classes of core biosynthetic enzymes in fungal BGCs are non-ribosomal peptide synthetases, PKS, and terpene synthases [4, 26, 29]. These enzymes are known to biosynthesize a variety of antibiotics and immunosuppressants with pharmaceutical potential. Thus, they have become popular targets for natural product discovery [29–31].

The investigations of BGCs confirmed the presence of diverse untapped fungal natural products [32–34]. As a growing number of lichen genomes increased, the identification of BGCs linked to the biosynthesis of lichen natural products also increased. To date, BGCs for atranorin [12] and lecanoric acid [9] were identified and validated by heterologous expression. The BGCs of biruloquinone [21], grayanic acid [10], and usnic acid [35, 36] were identified with transcriptional evidences. In addition, the BGCs for gyrophoric acid [37], olivetoric acid/ physodic acid [38] were tentatively assigned via phylogenetic analysis. Despite a great deal of natural products from fungal lineages [2, 26, 39, 40], BGCs responsible for the biosynthesis of major lichen metabolites are still unknown [41] due to extremely slow growth rate of mycobionts in culture [8] and limited molecular tools for manipulating mycobionts recalcitrant to genetic transformation [12]. To overcome these hurdles in studying on biosynthetic genes in lichens, the expression levels of biosynthetic genes were correlated with the amount of produced metabolites of interest [10, 21, 42]. Genome-guided gene discovery and metatranscriptomic analysis have aided the identification of BGCs for metabolite of interest in lichens, indicating that the number of BGCs found in fungal genomes outnumber the known fungal products they produce [7, 22, 28, 35, 36]. In this study, we performed transcriptome analysis of *C*. *metacorallifera* growing on growth media containing different carbon sources and singled out a candidate biosynthetic gene cluster linked to cristazarin production. A polyketide pathway for the biosynthesis of a naphthazarin derivative, cristazarin, was proposed based on putative function of tailoring enzymes and phylogenetic placement of the polyketide synthase in the cristazarin BGC.

## Materials and methods

### Fungal isolate and growth conditions

*Cladonia metacorallifera* KoLRI002260 was obtained from the Korean Lichen and Allied Bioresource Center (KOLABIC) of the Korean Lichen Research Institute (KoLRI) at Sunchon National University, Korea [16]. The fungal isolate was grown in malt-yeast agar medium (MYA; BD Biosciences, Baltimore, MD, USA) for about 2 months, and agar plugs containing mycelia were gently crushed using a sterile mortar and a pestle. One hundred microliter of homogenized fungal suspension was inoculated onto MYA or Lilly and Barnett’s (LB) liquid medium (10 g carbon sources, either glucose, fructose, ribitol, or sorbitol; 2 g asparagine; 1 g KH_2_PO_4_; 0.5 g MgSO_4_·7H_2_O; 0.2 mg Fe(NO_3_)_3_·9H_2_O; 0.2 mg ZnSO_4_·7H_2_O; 0.1 mg MnSO_4_·4H_2_O; 0.1 mg thiamine; and 5 μg biotin in 1 L of distilled water) [43], which were overlaid with a sterilized 0.45 μm pore cellulose nitrate membrane (Whatman, Cytiva, Marlborough, MA, USA). The cultures were incubated at 15 ˚C under fluorescent light (6500 k, 18 wattages).

### Genome annotation and biosynthetic gene cluster identification

The genome assembly of *C*. *metacorallifera* was annotated using the GenSAS (v.6.0) annotation pipeline [44]. In brief, low-complexity regions and repeats were masked using RepeatModeler (v1.0.11) and RepeatMasker (v4.0.7), setting the DNA source to ‘Fungi’. A masked consensus sequence was generated, on which *ab initio* gene prediction was performed using the following tools: (i) Augustus (v3.3.1), selecting *A*. *nidulans* as a trained organism; (ii) GeneMark-ES (v4.33); (iii) Genscan (v1.0), using a parameter setting for Human and other vertebrates; and (iv) GlimmerM (v2.5.1), selecting *Aspergillus* as a trained organism. For homology-based predictions, the NCBI reference transcript and protein databases for Fungi were searched, using (v) BLAST+ (v2.7.1) and (vi) DIAMOND (v0.9.22), respectively. For the consensus gene model prediction using EVidenceModeler (v06-25-2012), the above-mentioned standalone gene predictions were weighted as follows: (i) five, (ii) ten, (iii) one, (iv) one, (v) five, and (vi) five. A total of 10,828 open reading frames were predicted in the current genome annotation (S1 Table). For BGC identification, the genome assembly and gene annotation files of *C*. *metacorallifera* were processed by the antiSMASH program (v5.01), with the parameter setting “–minimal” [45].

### Identification of syntenic gene clusters

Genome assemblies of *Cladonia* species were downloaded from the Joint Genome Institute (JGI) or the National Center for Biotechnology Information (NCBI): *C*. *borealis* (NCBI accession: JAFEKC000000000), *C*. *grayi* (JGI accession: Cgr/DA2myc/ss v2.0), *C*. *macilenta* (NCBI accession: GCA_000444155.1), *C*. *rangiferina* (NCBI accession: GCA_006146055.1), and *C*. *uncialis* (NCBI accession: GCA_002927785.1). The genome assemblies were annotated using the GenSAS pipeline, and BGCs were mined from the five related *Cladonia* spp., using antiSMASH, as described above. To search for homologous BGCs in the six C*ladonia* spp., we used the BiG-SCAPE program which is useful for investigating the conservation and variation of BGCs in related species. Based on the Jaccard index of domain types, domain sequence similarity, and domain adjacency index, the BiG-SCAPE program calculates a similarity matrix between pairwise combinations of clusters where smaller values indicate greater BGC similarity. A cutoff value of 0.5 was used to identify homologous gene clusters.

### RNA extraction for RNA-seq

Red pigment produced by *C*. *metacorallifera* was observed in LB media containing either glucose or fructose 3 weeks after the incubation. Mycelia were harvested by scraping fungal mass growing on the membranes with a razor blade, and ground to a fine powder in liquid nitrogen. Total RNA was extracted using an easy-spin total RNA extraction kit (iNtRON Biotechnology, Seoul, Korea). cDNA libraries were constructed, using the TruSeq RNA library preparation kit (San Diego, CA, USA), and sequenced on the HiSeq2000 platform at Macrogen Inc. (Seoul, Korea). Raw reads (paired-end, 100 bp) were further processed and filtered, using the TrimGalore (v0.6.6) (https://www.bioinformatics.babraham.ac.uk/projects/trim_galore/). Filtered reads were mapped to the genome sequence of *C*. *metacorallifera*, using the HISAT2 program (v2.1.0). Mapped reads on genomic features, such as exon and intron, were calculated, using the htseq-count program. Gene expression levels in reads per kilobase per million mapped reads (RPKM) values were computed and normalized by effective library size estimated by trimmed mean of M values, using the edgeR R package (v3.26.8).

### Phylogenetic analysis

Protein sequences for 54 PKS genes related to melanin biosynthesis were downloaded from National Center for Biotechnology Information (NCBI) (S2 Table). The KS domain sequences of the 54 PKS were extracted in the NaPDoS webserver [46, 47], and were aligned using MAFFT (v7.310) [48] with the ‘auto’ setting (S3 Table). Poorly aligned regions of the resulting multiple sequence alignment were trimmed, using the Trimal program, with the parameter setting “–gappyout” [49]. A maximum likelihood tree was constructed using the RAxML program (v8.2) [50] and annotated using iTOL (v5.7) [51]. Nodal supports were evaluated by 1,000 bootstrap replications.

### Ethical statement

This research did not involve human participants and/or animals.

## Results

### Gene expression profiling of PKS genes in *Cladonia metacorallifera*

The mycobiont isolated from *C*. *metacorallifera* exhibited carbon source dependency in its chemical profile, producing cristazarin on fructose, but not on sugar alcohols and other carbon sources [14]. This prompted us to examine PKS expression profiles in the mycobiont growing on different carbon sources to identify a BGC involved in cristazarin production. First, we identified PKS genes present in the genome of the *C*. *metacorallifera* mycobiont. Compared to the previous annotation [16], the newly annotated genome contained 10,818 protein-coding genes, among which 39 genes encode iterative type I PKS. The PKS genes included *PKS1*, *PKS2*, *PKS3*, *PKS5*, *PKS7*, *PKS10*, *PKS11*, *PKS13*, *PKS15*, and *PKS16*, which were originally described in an earlier study on the *Cladonia chlorophaea* species complex [52]. Among these previously annotated PKSs, *PKS1* was predicted to be involved in depside- and depsidone-class metabolites derived from 3-methylorsellinic acid [12, 53]. Also, *PKS13* and *PKS15* were phylogenetically related to PKS genes involved in melanin production [12]. Notably, *PKS8*, *PKS16*, and *PKS21* were previously connected to the biosynthesis of usnic acid [29, 35], grayanic acid [10], gyrophoric acid [37], lecanoric acid [9], olivetoric acid, physodic acid [38], and biruloquinone [21]. However, there were still many undescribed PKS genes in the genome of the *C*. *metacorallifera* mycobiont.

**Fig 1:**
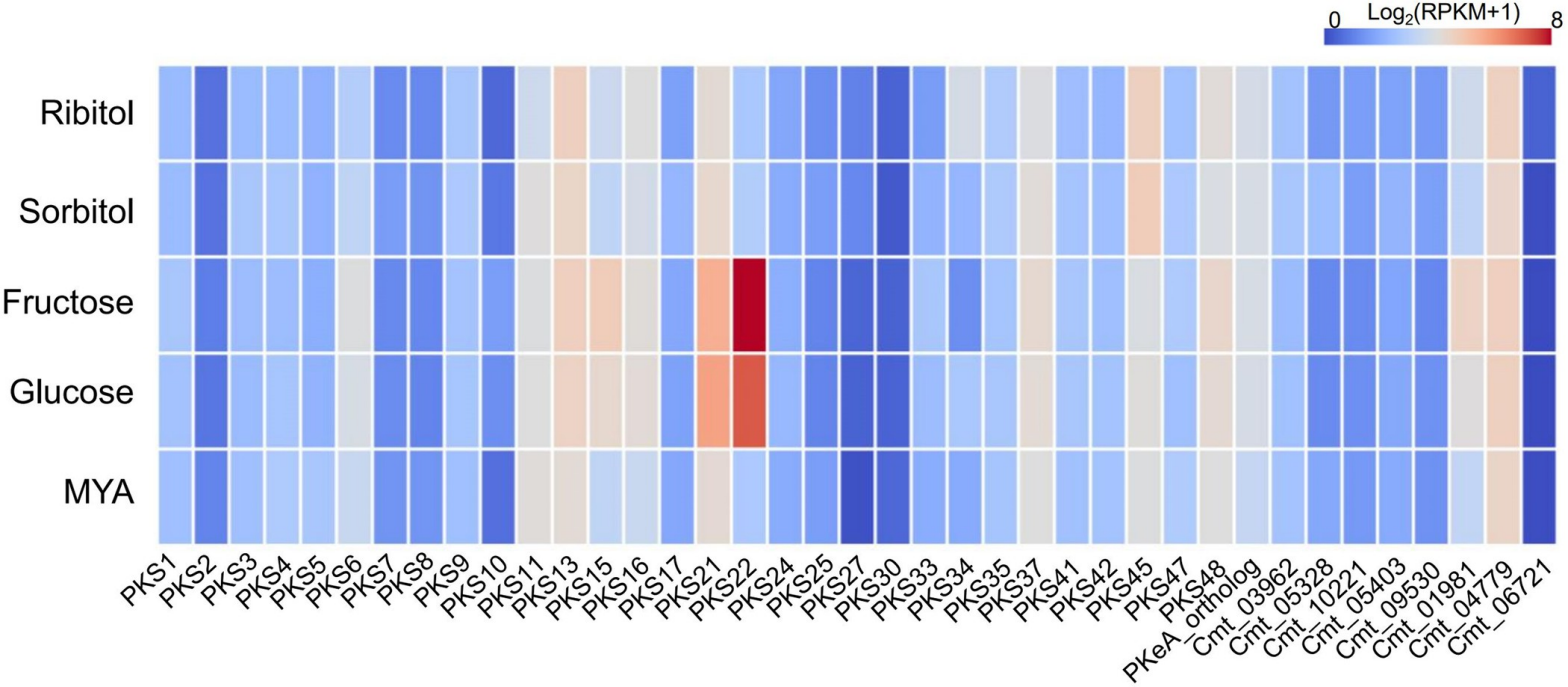
Identification of polyketide synthase for the biosynthesis of cristazarin. PKS gene expression profiles of the *Cladonia metacorallifera* mycobiont grown on culture media containing either fructose, glucose, ribitol or sorbitol as a single carbon source and on malt extract agar (MYA). Expression values (Log2-transformed (RPKM+1)) are shown as heat maps for 39 PKS genes in *C*. *metacorallifera*.

Previously, production of cristazarin was induced when the *C*. *metacorallifera* mycobiont is grown on media containing fructose as a sole carbon source after 3 weeks of incubation [14]. Therefore, expression levels of PKS genes in the *C*. *metacorallifera* mycobiont grown in culture media containing different sugars as a sole carbon source were compared to identify a PKS gene responsible for cristazarin production at 3 weeks after incubation. Among 39 iterative type I PKS genes, only *PKS22* was highly upregulated in growth media containing fructose, and, to a lesser extent, it was also upregulated in a glucose-containing media (Fig 1). On the other hand, expression levels of *PKS22* remained basal in MYA and growth media containing sugar alcohols, such as ribitol and sorbitol (Fig 1), with which *C*. *metacorallifera* was not able to produce cristazarin [14]. This indicated that *PKS22* is likely involved in the biosynthesis of cristazarin.

### Demarcation of boundaries for the cristazarin BGC

**Fig 2:**
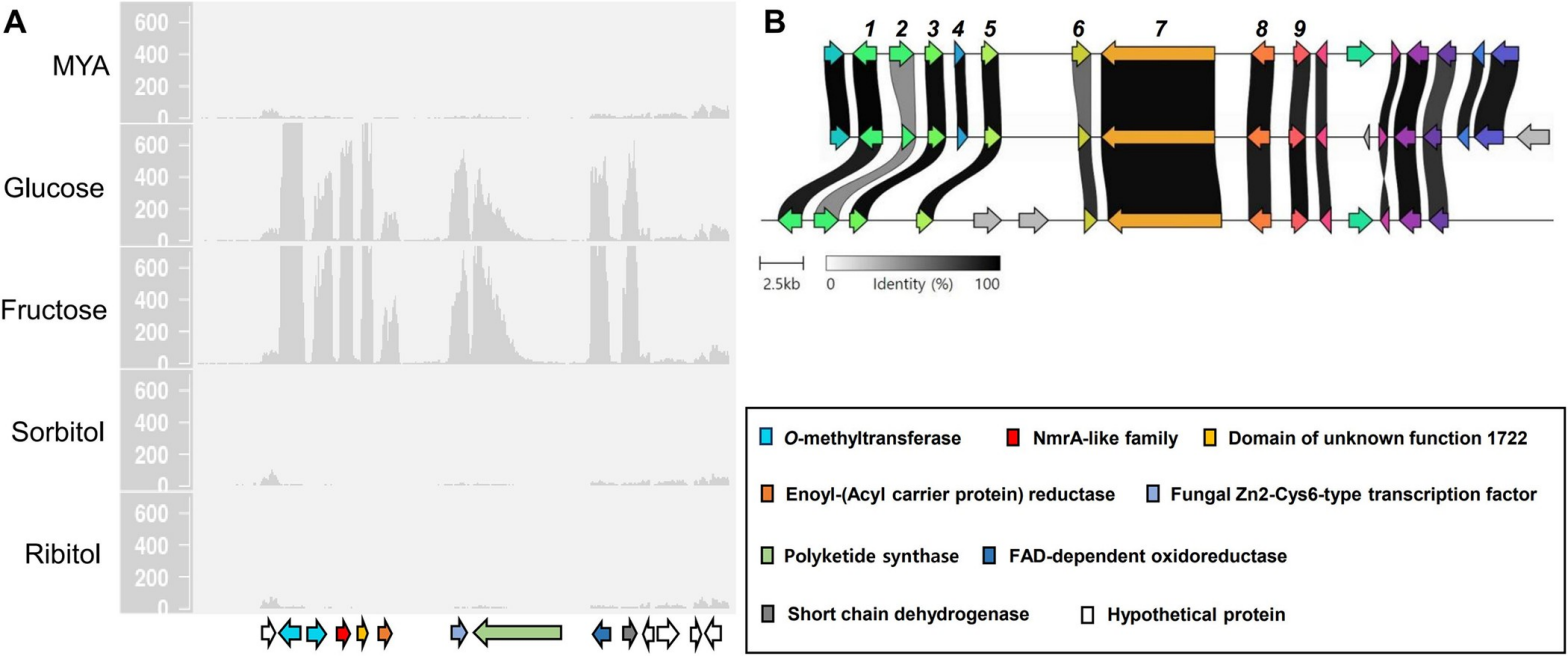
Demarcation of the cristazarin BGC boundaries by mapped reads of RNA-seq. (A) Mapped reads of five RNA-seq samples of the *C*. *metacorallifera* mycobiont growing on different carbon sources were shown for a genomic locus harboring the PKS22 (*crz7*; gene ID: Cmt_01711). RNA-seq reads mapped on the *C*. *metacorallifera* reference genome were subsampled to 60 million reads for visual comparison of expression levels between samples. Arrows on the x-axis indicate genes (Cmt_01704–Cmt_01717). The numbers on the y-axis are per-base coverage of mapped reads. (B) Synteny of the cristazarin BGCs in *Cladonia* species: *C*. *metacorallifera* (top), *C*. *macilenta* (middle), and *C*. *borealis* (bottom). Arrows indicate open reading frames (ORFs) found in the BGCs, and homologous genes were represented with different colors. Numbers above the arrows indicate *crz1*–*crz9*.

To delimit the BGC borders and identify genes responsible for the whole biosynthetic pathway of cristazarin, mapped RNA-seq reads was visualized on a genomic locus harboring *PKS22*. We identified several biosynthetic genes and transcription factor-like genes, which were co-expressed with *PKS22* in *C*. *metacorallifera* (Fig 2A). As with *PKS22*, these genes were specifically induced in growth media containing fructose or glucose as a sole carbon source, suggesting their involvement in the biosynthesis of cristazarin. These co-expressed genes included two *O*-methyltransferase genes (*crz1* and *crz2*), a gene encoding NmrA-like family (*crz3*), a gene harboring a conserved domain of unknown function 1722 (*crz4*), a tetrahydroxynaphthalene reductase-like gene (*crz5*), a fungal-specific transcription factor (*crz6*), a FAD-dependent oxidoreductase gene (*crz8*), and a short chain dehydrogenase gene (*crz9*) (Table 1; S4 Table).

**Table 1:** Annotation of the cristazarin biosynthetic gene cluster in *Cladonia metacorallifera*.

Gene ID (Cmt)	Gene name	Conserved domain (accession)	*E*-value
01705	*crz1*	*O*-methyltransferase (PF00891)	7.35e–17
01706	*crz2*	*O*-methyltransferase (PF00891)	1.72e–12
01707	*crz3*	a transcriptional regulator NmrA-like family (PF05368)	7.50e–17
01708	*crz4*	Domain of unknown function 1772 (PF08592)	4.75e–21
01709	*crz5*	Enoyl-(acyl carrier protein) reductase (PF13561)	2.33e–50
01710	*crz6*	GAL4-like fungal Zn2-Cys6 binuclear cluster domain (PF00172)	1.78e–08
01711	*crz7*	Polyketide synthase, PKS22 (SAT-KS-AT-PT-ACP-ACP-TE)^*a*^	N/A
01712	*crz8*	FAD-dependent oxidoreductase (COG0654)	5.75e–27
01713	*crz9*	Short chain dehydrogenase (PF00106)	2.84e–24

^*a*^The polyketide synthase domain architecture of PKS22. SAT, starter unit ACP transacylase; KS, keto synthase; AT, acyl transferase; PT, product template; ACP, acyl-carrier protein; TE, thioesterase. N/A = not applicable.

*PKS22* (hereafter referred to as *crz7*) homologs can be found in related *Cladonia* spp., such as *C*. *borealis*, *C*. *macilenta* and *C*. *uncialis* [12]. Thus, we searched for homologous BGCs in available genomes. BGCs highly syntenic to the cristazarin BGC in *C*. *metacorallifera* were found in the genomes of *C*. *borealis* and *C*. *macilenta* (Fig 2B). All the co-expressed genes were found in *C*. *borealis* and *C*. *macilenta*, suggesting genetic potentials to produce cristazarin in these closely-related species. Although there is a *crz7* homolog in the genome of *C*. *uncialis*, it was found in a very short contig including no additional gene, indicating that *C*. *uncialis* may have lost the ability to produce cristazarin. To investigate the presence of a PKS22 homolog in other lichen species, we blasted the amino acid sequence of PKS22 in *C*. *metacorallifera* against the NCBI database. Although there are many genome sequences of Lecanorales available in NCBI database, the best hits for PKS22 were PKSs found in the Teloschistales, which showed protein sequence identities ranging from 59–68% to the PKS22. In the current database (accessed on June 05, 2023), we were unable to detect a PKS22 homolog in the Lecanorales, to which the genus *Cladonia* belong.

### Phylogenetic dereplication of the biosynthetic pathway for cristazarin

Crz7 is closely related to the non-reducing PKS (NR-PKS) group II [12], in which many PKSs are known to be involved in the biosynthesis of 1,3,6,8-tetrahydoxynaphthalene (T4HN) or 2-acetyl-1,3,6,8-tetrahydoxynaphthalene (AT4HN) that are further processed to dihydroxynaphthalene (DHN) and polymerized to melanin pigment [54–58]. To predict the polyketide scaffold biosynthesized by Crz7, we reconstructed a phylogenetic tree of many previously characterized group II NR-PKSs, as well as NR-PKSs belonging to groups III, V, and XI that form outgroups to group II (S2 Table) [12, 59].

**Fig 3:**
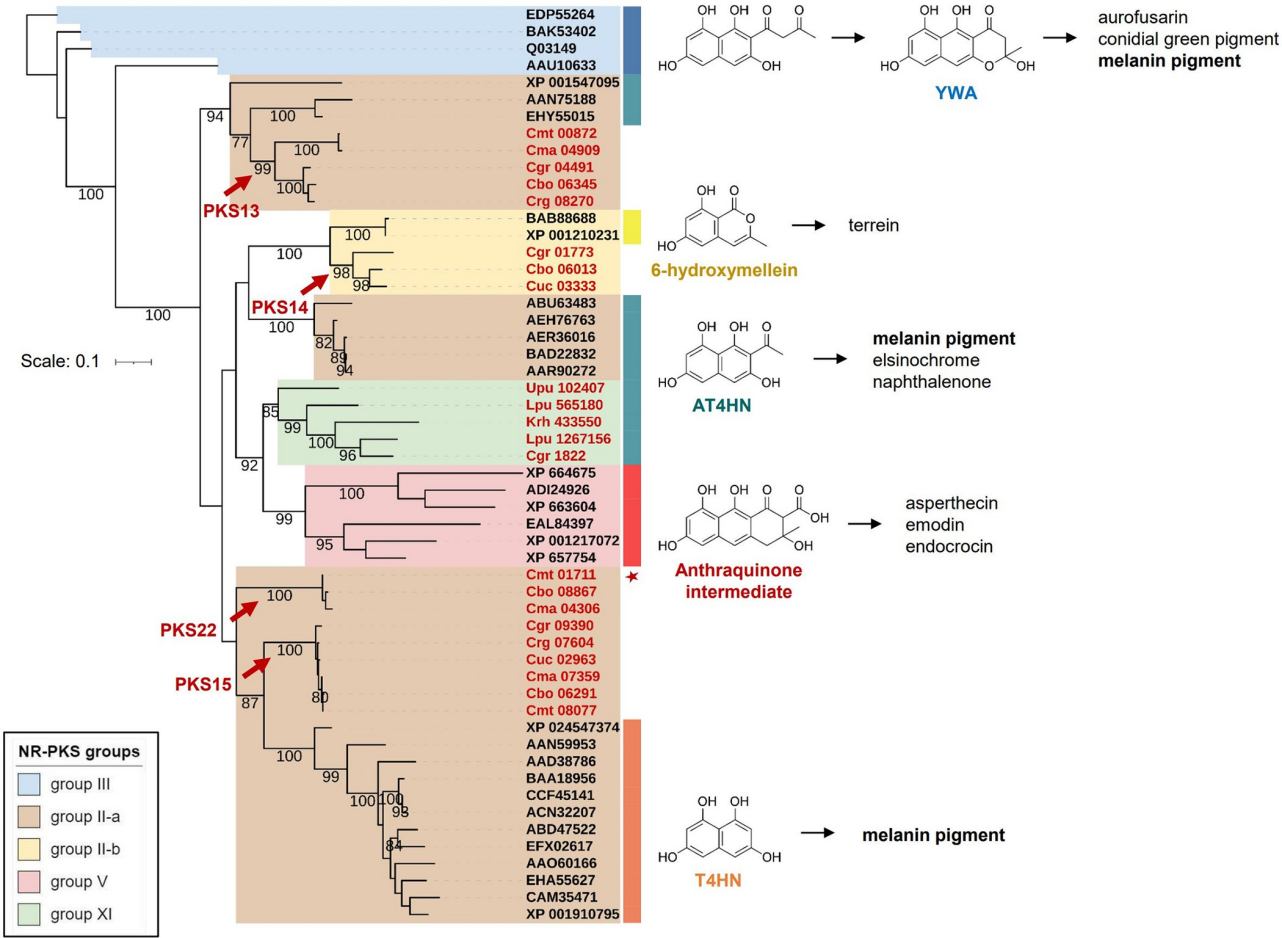
Phylogenetic dereplication of polyketide synthases related to melanin production. A maximum likelihood phylogenetic tree of non-reducing polyketide synthases (NR-PKSs). Colored strips on the right side of leaves indicate polyketide backbones produced by the functionally characterized NR-PKSs. Clades of the previously described NR-PKS groups (Kim et al., 2021; Mosunova et al., 2022) were shaded with different colors (see inset). Arrows indicate clades of the *Cladonia* PKS families, PKS13, PKS14, PKS15, and PKS22, that belong to group II NR-PKS (Kim et al., 2021). Leaves highlighted in red are lichen NR-PKSs. Crz7 in *C*. *metacorallifera* was denoted by a red asterisk. An NR-PKS in *Aspergillus fumigatus* (EDP55264) that produce melanin pigment using YWA as a precursor was set as an outgroup. Bootstrap values of greater than 75% were shown. Branch lengths are proportional to the inferred amount of evolutionary change, and the scale represents 0.1 amino acid sequence substitutions per site. T4HN, 1,3,6,8-tetrahydroxynaphthalene; AT4HN, 2-acetyl-1,3,6,8-tetrahydroxynaphthalene.

Although phylogenetic relationships between the subclades of NR-PKS group II were uncertain with low bootstrap supports, the PKS22 family including the Crz7 formed a highly-supported clade distinct from, but closely-related to the NR-PKS group II-a (Fig 3). The newly described NR-PKS group XI involved in the biosynthesis of naphthalenone formed a distinct clade sister to the NR-PKS group V involved in the biosynthesis of anthraquinone-class metabolites (e.g. asperthecin, emodin, and endocrocin). It has been shown that most of the groups II-a and XI NR-PKSs biosynthesize hexaketide products, AT4HN and T4HN [54, 59–63]. Given the close phylogenetic distance of Crz7 to group II-a PKS producing AT4HN and T4HN, predicted product of Crz7 is likely AT4HN or T4HN. The PKS15 family in *Cladonia* spp. is placed sister to the NR-PKS group II-a that are known to be able to directly biosynthesize T4HN as a precursor for melanin production through their deacetylase activity [55], while the NR-PKSs placed sister to the PKS13 family in *Cladonia* spp. are known to first biosynthesize AT4HN and also require an additional hydrolase activity (Ayg1) to biosynthesize T4HN for melanin production [56, 64] (Fig 3). These two functionally-related PKSs, PKS13 and PKS15 in *C*. *metacorallifera* showed protein sequence identity of 47%, and the Crz1 (PKS22) exhibited 50% sequence identity to PKS13 and PKS15 (S2 Table).

### A proposed biosynthetic pathway for cristazarin

**Fig 4:**
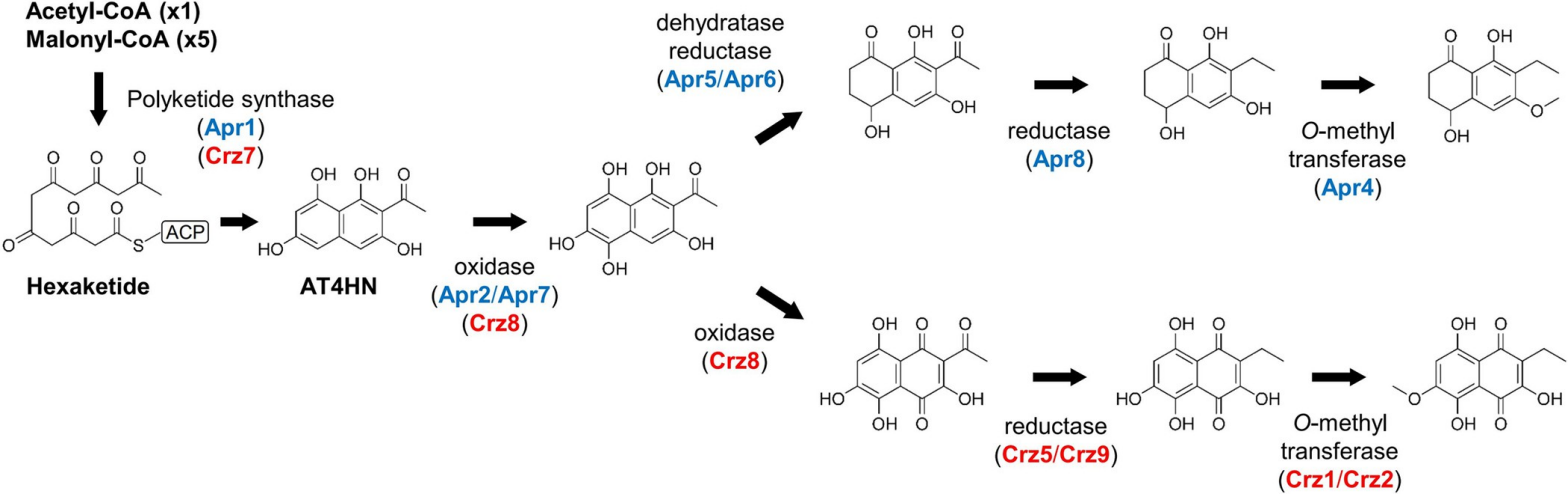
Divergent biosynthetic routes of cristazarin. A proposed biosynthetic pathway of cristazarin from a polyketide precursor, 2-acetyl-1,3,6,8-tetrahydoxynaphthalene (AT4HN), can be deduced from putative function of tailoring enzymes in the cristazarin gene cluster (red). Note that a biosynthetic pathway for 6-*O*-methylasparvenone (a naphthalenone) is analogous to that for cristazarin, involving several biosynthetic genes in the naphthalenone BGC (blue) in *Aspergillus parvulus* (Mosunova et al. 2022). ACP, acyl carrier protein domain.

The phylogenetic placement of Crz7 proximal to PKSs known to produce AT4HN and T4HN suggested that the polyketide product of Crz7 is likely AT4HN, from which a probable biosynthetic route of cristazarin can be deduced (Fig 4). In many fungi, melanin pigment is also biosynthesized using AT4HN or YWA (a naphthopyrone compound) that is further processed by a hydrolase (such as Ayg1 in *Aspergillus fumigatus*), resulting in T4HN [54, 56]. Some group II NR-PKSs have a deacetylase activity, and are able to produce T4HN without the aid of a hydrolase [55]. The presence of two *O*-methyltransferase (*crz1* and *crz2*), a enoyl reductase (*crz5*), an oxidase (*crz8*), and a short-chain dehydrogenase (*crz9*) encoded in the cristazarin BGC is consistent with methylation of a hydroxyl group, addition of two hydroxyl groups to the naphthalene core ring, and reduction of the acetyl side chain (Fig 4). The *crz4* encoding a conserved domain of unknown function showed a co-expression pattern with the other biosynthetic genes (Fig 2). The proposed biosynthetic route of cristazarin is analogous to, with a minor difference, that of naphthalenones whose biosynthetic gene cluster was recently identified in *A*. *parvulus* [59] (Fig 4).

## Discussions

Lichen-forming fungi are known to produce a large number of secondary metabolites [36, 65]. To date, only the two BGCs for atranorin [12] and lecanoric acid [9] were identified by heterologous expression. In addition, a few lichen natural products have been connected to BGCs including biruloquinone [21], grayanic acid [10], and usnic acid [35, 36] with transcriptional evidence. The BGCs of gyrophoric acid [37], olivetoric acid/ physodic acid [38] were tentatively assigned via phylogenetic analysis. antiSMASH, BiG-SCAPE, BiG-FAM, and MiBiG, assisted the in silico identification of candidate BGCs for the production of lichen metabolites [19, 22, 27, 45, 66]. Although putative lichen BGCs have been identified using these programs and databases, heterologous expression validates gene function as seen in atranorin [12] and lecanoric acid [9].

The lichen-derived red pigment, cristazarin, was first discovered from *in vitro* culture of *C*. *cristatella* [13]. Other naphthazarin derivatives structurally-related to cristazarin were an orange pigment, squamarone, and its derivatives isolated from a lichen *Squamarina cartilaginea* [67]. It is noteworthy that there are multiple methoxy groups in the core naphthazarin ring of squamarone, which are likely catalysed by more than one *O*-methyltransferase. The degree of methylation in hydroxyl groups of the core naphthazarin ring appears to account for the colour difference of cristazarin (red) and squamarone (orange), similar to what is observed in antocyanins [68]. Despite only one methoxy group in cristazarin, there are two *O*-methyltransferase (*crz1* and *crz2*) present in the cristazarin BGC, indicating that the two genes play a redundant function or one of them is non-functional. Although no BGC has been reported in *Squamarina cartilaginea*, one can predict that the as-yet-unknown squamarone BGC has multiple functional *O*-methyltransferases. Identification and functional characterization of such *O*-methyltransferases may enable production of customized colorants derived from a naphthazarin backbone.

Jeong et al (2021) determined growth condition that produce cristazarin in axenic culture of the mycobiont and performed expression analysis of candidate PKS genes [14]. In this study, we performed RNA-seq to obtain global gene expression profiles in different nutrient conditions and singled out a PKS that is most likely involved in the biosynthesis of cristazarin. The combining transcriptome analysis and chemical profiling in inducing- and non-inducing conditions identified the cristazarin BGC in *C*. *metacorallifera*. *Crz7* was highly upregulated in growth media containing fructose or glucose. The second most upregulated gene was *PKS21*, an ortholog of the *brq5* involved in the biosynthesis of biruloquinone, a rare phenanthraquinone-class metabolite, which we previously identified in *C*. *macilenta* [21]. However, biruloquinone has never been detected in culture extracts of *C*. *metacorallifera*, indicating a high level of PKS gene expression is required to produce final polyketide products above the detection limit of analytical devices [69–71]. The highly oxygenated bicyclic core of cristazarin suggested that the cognate PKS should be an NR-PKS. Thus, we can rule out 22 reducing type PKS, including *PKS48* that showed slight upregulation in fructose- and glucose-containing media. Also, *PKS15* is upregulated in fructose- and glucose-containing media. The BGCs including *PKS15* and *PKS13* contain both scytalone dehydratase and T4HN reductase. The two hallmark enzymes for melanin production and close phylogenetic relationships of *PKS15* to other previously characterized melanin PKSs enabled us to rule out *PKS15* as a candidate for cristazarin PKS.

The proposed biosynthetic pathway of cristazarin seems to be analogous to that of naphthalenone. However, a reciprocal best hit BLAST analysis indicated that none of the *crz* genes encoding tailoring enzymes are best hits of the *apr* genes in the naphthalenone BGC, and vice versa (S5 Table). Apr2 and Apr7 have a protein domain COG0277 (FAD/FMN-containing dehydrogenase), while Crz8 has a protein domain COG0654 (an UbiH domain related to FAD-dependent oxidoreductase). It is conceivable that these enzymes are involved in oxidation of the AT4HN backbone, albeit they seem not related to each other, showing low sequence identify. In the naphthalenone biosynthetic pathway, Apr8, an aldo-keto reductase, was hypothesized to play a role in reduction of the carbonyl group. In the cristazarin BGC, there was no such an enzyme. Instead, Crz9 and Crz5 are likely involved in the successive reduction processes: carbonyl to enoyl by Crz9 (a short chain dehydrogenase), and then enoyl to the fully saturated side chain by Crz5 (an enoyl reductase). The biosynthetic logic of cristazarin can be deduced from the putative function of the tailoring enzymes.

GAL4-type transcriptional regulators with a Zn2-Cys6 binuclear cluster domain play key roles in either activating or repressing the whole BGC in a pathway-specific manner [72, 73]. The high expression levels of *crz6* encoding a Zn2-Cys6 transcription factor in inducing conditions indicated that the *crz6* is a positive regulator for cristazarin production. *Crz3* encodes an NmrA-like family protein and showed highly correlated expression pattern with the other biosynthetic genes in the cristazarin BGC. In other fungi such as *A*. *nidulans*, *Neurospora crassa*, and *Fusarium fujikuroi*, NmrA acts as a repressor for the AreA activity that is important for activation of genes involved in nitrogen catabolism [74]. Therefore, *crz3* may play a role in balancing primary and secondary metabolism to favor the biosynthesis of cristazarin over other metabolites. An *nmrA*-like gene also can be found in the naphthalenone BGC in *A*. *parvulus* [59], suggesting its conserved regulatory role in production of asparvenone and cristazarin that are biosynthesized by an analogous pathway.

Fungal pigments are used as natural colorants in industries, many of which are of polyketide origin [75, 76]. Each group of NR-PKS produces distinct and characteristic chemical scaffolds for fungal pigments: group II producing AT4HN or T4HN for melanin, group III and IV producing naphthopyrones, group V producing anthraquinones, and group VII producing azaphilones. Crz7 and Apr1 produce cristazarin and asparvenone from AT4HN, respectively, through an analogous pathway. While the NR-PKS group XI (Apr1) and group V were placed as sister taxa with strong bootstrap support, Crz7 was phylogenetically related to group II NR-PKSs for melanin production. In fungi, melanin pigment is biosynthesized from AT4HN and YWA by NR-PKSs group II and group III, respectively [57]. In *Botrytis cinerea*, BcPKS12 (related to the PKS13 family in *Cladonia* spp.) and BcPKS13 (related to the PKS15 family), are developmentally-regulated and responsible for melanization of sclerotia and conidia, respectively [58]. Tissue-specific regulations of different pigment systems are also observed in perithecial wall pigments in *Fusarium* species [77, 78]. The role of the PKS13 and PKS15 NR-PKS families in lichens are currently unknown, however it may have evolved to have tissue-specific roles during the life cycle of lichens.

The group II NR-PKSs often exhibit promiscuity with respect to polyketide chain length:TerA producing tri-, tetra-, and penta-ketide [79], Apr1 producing penta- and hexa-ketide [59], and PksA producing hexa- and hepta-ketide [57], when they are heterologously expressed. This chain length promiscuity of PKSs may be one of the sources of diversification driving chemical evolution. Nonetheless, most of group II NR-PKSs produce a hexaketide product AT4HN in natural hosts, except for the group II-b NR-PKSs including the PKS14 family in *Cladonia* species. The group II-b NR-PKSs are known to biosynthesize a pentaketide product, 6-hydroxymellein [35, 79]. Many of the group II NR-PKSs for melanin production have evolved to have a deacetylase activity so that THN can be generated from AT4HN without the help of a separate hydrolase enzyme, such as Ayg1 [55]. In contrast, Crz7 and Apr1 do not possess such an activity and may have contributed to establishing diverse pigment systems in fungi.

## Conclusions

Lichens are treasure chests for novel secondary metabolites. In this study, we linked a putative biosynthetic gene cluster to cristazarin via transcriptome analysis and phylogenetic dereplication. Cristazarin BGCs were specifically found in lichens, and the PKS22 family including Crz7 formed a distinct clade from related NR-PKS in other non-lichenized fungi. The identification of the putative cristazarin BGC will be an important addition to the fungal natural product BGC database, as this is the first report linking a BGC to a naphthazarin-class metabolite. Given the frequent appearance of unexpected metabolites from lichen mycobionts [13, 21, 80–82], transcriptome and phylogenetic analyses of core biosynthetic genes, such as PKS, are an efficient way to connect BGCs to novel metabolites of interest in lichen mycobionts that are recalcitrant to genetic transformation for functional studies of biosynthetic genes.

## Supporting information

S1 Table The current genome annotation of *C*. *metacorallifera*. (FAA)

S2 Table Protein sequences for the 54 PKS genes related to melanin biosynthesis. (XLSX)

S3 Table The KS domain sequences of the 54 PKS genes related to melanin biosynthesis. (FAA)

S4 Table The NCBI accession of the sequences of cristazarin BGC in *C*. *metacorallifera*. (TXT)

S5 Table Reciprocal best hit BLAST analysis of the members of the cristazarin and naphthalenone BGCs. (DOCX)
