# Supplementary material for: Identification of a biosynthetic gene cluster for a red pigment cristazarin produced by a lichen-forming fungus Cladonia metacorallifera
Source: PLoS One. 2023 Jun 23;18(6):e0287559. doi: 10.1371/journal.pone.0287559 (PMC10289310; doi:10.1371/journal.pone.0287559)
Supplement: S5 Table — (DOCX) [file pone.0287559.s005.docx]

| **Query** | **Best hit (% identity)** | **Hit to Apr or Crz (% identity)** |
| --- | --- | --- |
| Crz1 | 192783^a^ (39) | Apr4 (36) |
| Crz2 | 192783^a^ (32) | Apr4 (28) |
| Crz3 | 205944^a^ (43) | no hit |
| Crz4 | 211188^a^ (26) | no hit |
| Crz5 | 200126^a^ (52) | Apr6 (39) |
| Crz6 | 201405^a^ (27) | no hit |
| Crz7 | 81212^a^ (47) | Apr1 (47) |
| Crz8 | 184578^a^ (39) | no hit |
| Crz9 | 190631^a^ (46) | Apr6 (22) |
| Apr1 | Cmt_01711^b^ (47) | Crz7 |
| Apr2 | Cmt_07289^b^ (28) | no hit |
| Apr3 | Cmt_03371^b^ (26) | no hit |
| Apr4 | Cmt_03334^b^ (38) | Crz1/Crz2 |
| Apr5 | Cmt_10754^b^ (45) | no hit |
| Apr6 | Cmt_08079^b^ (56) | Crz5/Crz9 |
| Apr7 | Cmt_10394^b^ (32) | no hit |
| Apr8 | Cmt_07751^b^ (39) | no hit |

**Supplementary Table S5.** Reciprocal best hit BLAST analysis of the members of the cristazarin and naphthalenone BGCs.

^a^ Protein IDs of *Aspergillus parvulus* strain CBS 136.61 in JGI annotation, available from the Mycocosm website (<https://mycocosm.jgi.doe.gov/Aspparv1/Aspparv1.home.html>)

^b^ Protein IDs of *Cladonia metacorallifera* in GenSAS annotation, available in Supplementary Table S1
